# Development and validation of a nutritional literacy assessment scale for nasopharyngeal carcinoma patients undergoing concurrent chemoradiotherapy

**DOI:** 10.3389/fnut.2025.1589233

**Published:** 2025-10-16

**Authors:** Zhang Yanxin, Huang Xiaojun, Yang Guirong, Li Wei, Liang Limin, Wei Lina, Lu Jiamei

**Affiliations:** Department of Radiotherapy, The First Affiliated Hospital of Guangxi Medical University, Nanning, China

**Keywords:** nasopharyngeal carcinoma, synchronized radiotherapy, nutritional literacy, reliability, validity, scale

## Abstract

**Background:**

Nasopharyngeal carcinoma (NPC) is a head and neck malignancy highly prevalent in East and Southeast Asia, for which concurrent chemoradiotherapy (CCRT) is the standard treatment option. However, the superimposed effects of radiotherapy (especially head and neck radiotherapy) and chemotherapy often lead to severe acute toxic reactions, insufficient nutritional knowledge of patients, and dietary misconceptions all affect the patient’s ability to eat and their nutritional status. Therefore, there is an urgent need to develop a Nutritional Literacy Scale (NLS) for patients undergoing simultaneous radiotherapy and chemotherapy for nasopharyngeal carcinoma (NPC) to optimize the overall nutritional management of NPC patients and to improve the therapeutic effect.

**Methods:**

The first draft of the scale was formed through literature analysis, semi-structured interviews, and expert correspondence. From April 2024 to December 2024, 245 patients with nasopharyngeal carcinoma treated with simultaneous radiotherapy and chemotherapy in the radiotherapy department of the First Affiliated Hospital of Guangxi Medical University were collected as the study subjects, and the scale was subjected to item analysis and reliability and validity tests, and the questionnaire was administered again to the patients 2 weeks later to measure the re-test reliability of the scale.

**Results:**

The Nutritional Literacy Scale for Nasopharyngeal Carcinoma Patients Undergoing Simultaneous Radiotherapy included 4 dimensions and 30 entries. Exploratory factor analysis extracted four male factors with a cumulative variance contribution of 62.3%; validated factor analysis showed that χ^2^/df = 1.155 (*p* = 0.085), GFI = 0.928, RMSEA = 0.025, CFI = 0.994, NFI = 0.956, and IFI = 0.994; questionnaire content validity I-CVI was 0.872 to 1.000, S-CVI was 0.932; Cronbach’s alpha coefficient for the total scale was 0.849, folded reliability was 0.869, and retest reliability was 0.960.

**Conclusion:**

The Nutritional Literacy Scale for Nasopharyngeal Cancer Patients Undergoing Simultaneous Radiotherapy has good reliability and validity.

## 1 Introduction

Nasopharyngeal carcinoma (NPC) is a prevalent head and neck malignancy in South China, with Guangxi Province exhibiting one of the highest global incidence rates (10–30 cases per 100,000 population) according to recent epidemiological data (1). Radiotherapy-based concurrent chemoradiotherapy remains the standard therapeutic regimen for NPC (2). However, this treatment frequently induces acute radiation reactions (e.g., nausea/vomiting, anorexia) and long-term complications (3, 4), compounded by tumor-related metabolic dysregulation, psychological distress, and nutritional misconceptions. These factors collectively impair patients’ nutritional beliefs and dietary intake patterns, ultimately leading to nutritional imbalance (5). Current evidence indicates that 30–80% of NPC patients develop malnutrition during treatment (6, 7), which not only reduces treatment tolerance resulting in therapy interruptions (8), but also adversely affects the quality of life, therapeutic efficacy, and long-term prognosis, imposing substantial socioeconomic burdens (9).

The Institute of Medicine (2004) defines health literacy as “the degree to which individuals can obtain, process, and understand basic health information needed to make appropriate health decisions” (10). As a critical subdomain of health literacy, nutrition literacy extends beyond mere nutritional knowledge to encompass the ability to acquire, interpret, and apply dietary information for informed decision-making (11, 12). Empirical studies demonstrate that nutrition literacy directly mediates dietary behaviors and nutritional status (13). This concept aligns with China’s national health strategies, including the “Healthy China 2030” Initiative and the National Nutrition Plan (2017–2030) (14), both emphasizing the pivotal role of nutritional education in population health improvement. For cancer populations, enhancing nutrition literacy represents an urgent clinical priority given its profound implications for treatment outcomes, survival duration, and quality of life.

Current nutrition literacy research predominantly focuses on pregnant women (15, 16), pediatric populations (17), caregivers (18), and hemodialysis patients (16). Notably, no validated assessment tool exists specifically for NPC patients undergoing chemoradiation. Existing generic instruments developed in Western populations demonstrate limited cross-cultural applicability due to significant dietary practice variations and disease-specific nutritional challenges (19). To address this gap, we developed an NPC-specific nutrition literacy scale grounded in the Information-Knowledge-Attitude-Practice (IKAP) theoretical framework (20). This study aims to: (1) establish a reliable assessment tool for evaluating nutritional literacy in NPC patients receiving chemoradiation; (2) characterize current nutritional literacy status and its determinants; (3) provide evidence-based insights for targeted interventions to optimize dietary behaviors and improve clinical outcomes.

## 2 Developing the scale

### 2.1 Establishment of the study team

**Figure 1:**
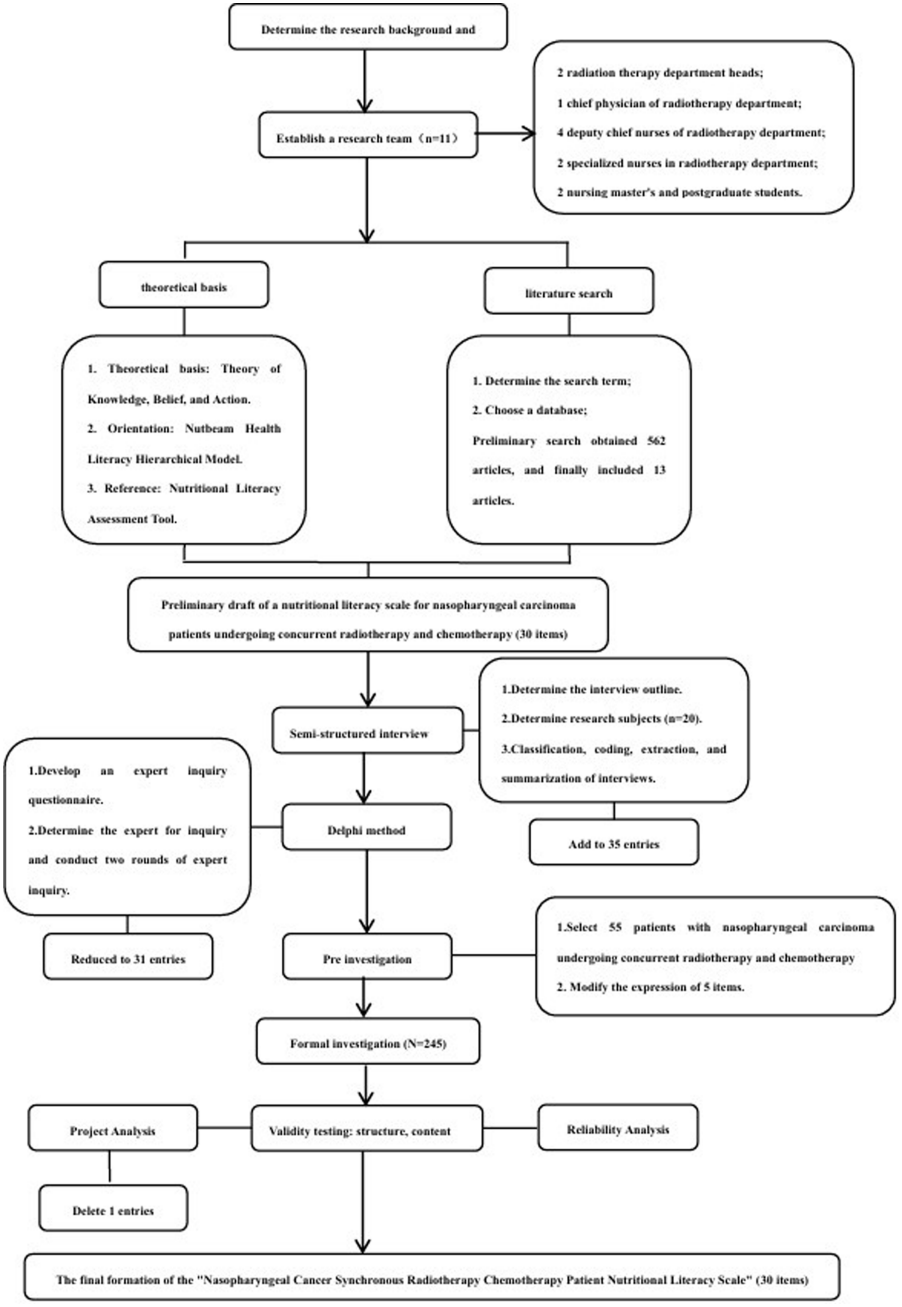
Consolidated standard of reporting trials (CONSORT) 2010 flow diagram.

The research team consisted of 2 radiotherapy department nursing chiefs, 1 radiotherapy department chief physician, 4 radiotherapy department deputy chief nurses, 2 radiotherapy department specialized nurses, 2 nursing master’s degree students, a total of 11 people. Members of the research team divided their work and responsibilities and were responsible for searching, reading, and analyzing the literature, formulating interview outlines, determining the interview subjects and conducting interviews, formulating the expert correspondence form based on the literature and interview results, selecting the experts, collating and analyzing the feedback results of each round of expert correspondence, determining whether it was necessary to add, modify or delete entries, and carrying out the clinical investigations and analyzing the data, etc. (see Figure 1).

### 2.2 Theoretical framework

Through reviewing domestic and international literature, this study takes “IKAP” (20) as the theoretical basis, Nutbeam health literacy hierarchical model (21) as the guide, and existing domestic and international nutrition literacy assessment instrument (NLit) (16, 22–25) as the reference, and combines the dietary and nutritional characteristics of nasopharyngeal carcinoma patients undergoing simultaneous radiotherapy to formulate the nutritional literacy framework for patients with nasopharyngeal carcinoma. Nutrition Literacy Assessment Instrument (NLit) (16, 22–25) was used as a reference, and the Nutrition Literacy Scale for NPC patients was developed by combining the characteristics of dietary and nutritional specialties of NPC patients undergoing simultaneous radiotherapy.

### 2.3 Literature search

With “nasopharyngeal cancer/nasopharyngeal cancer patients/nasopharyngeal cancer radiotherapy patients/oncology patients/cancer patients,” “diet or dietary or nutritional literacy/nutritional knowledge/nutritional behaviors/nutritional attitudes,” “questionnaire/questionnaire preparation “systematically searched Chinese databases such as China Biomedical Literature Database, Wipu Database, China Knowledge Network, Wanfang Database, etc.; with “Nasopharyngeal cancer/nasopharyngeal cancer patients/nasopharyngeal cancer Nasopharyngeal cancer/nasopharyngeal cancer patients/nasopharyngeal cancer radiotherapy and chemotherapy patients/tumor patients/cancer patients“”Dietary or dietary or nutritional literacy/nutritional knowledge/nutritional behavior/nutritional attitude” ‘Questionnaire/questionnaire development’ English keywords were searched in English databases such as PubMed, Web of Science, Embase, etc. to find studies related to the evaluation of nutritional literacy in nasopharyngeal cancer patients. Nutritional Literacy Evaluation in Nasopharyngeal Cancer Patients. The search period was from the establishment of the database to July 2023, and the preliminary search yielded 562 papers, and 13 papers were included after deleting duplicates and conference papers and reading the titles, abstracts, and full texts in turn. After the research team discussed and refined the contents of the literature one by one, a preliminary pool of entries containing 30 entries was formulated.

#### 2.3.1 Additional entries from qualitative interviews

##### 2.3.1.1 Determine the interview outline

Based on relevant literature (26), combined with clinical practice and the joint discussion of the research team, the interview outline of this study was formulated. Using the purposive sampling method, semi-structured interviews were conducted in July–August 2023 with patients with nasopharyngeal carcinoma treated for the first time with simultaneous radiotherapy in the First Affiliated Hospital of Guangxi Medical University.

(1) Do you think the tumor itself and its radiotherapy will have any effect on your nutritional status?
(2) What dietary and nutritional problems have you encountered during chemotherapy and how would you deal with them?
(3) In what way do you expect to learn about diet and nutrition and what nutritional knowledge would you like to learn?
(4) Will you follow what you have learned about diet and nutrition? Does it serve any purpose for you?

##### 2.3.1.2 Data collection methods

Face-to-face interviews were conducted by 2 research members after obtaining patients’ informed consent, 1 of whom was responsible for the interview and the other for the transcription and recording of the whole process, listening carefully during the interview, asking follow-up questions at the right time, avoiding induced questions, and recording non-verbal behavioral conditions and other information in the field. The criterion for the end of data collection was the occurrence of repeated information in the interview, i.e., the criterion of data saturation to determine the sample size, and 20 cases were finally included. Audio and note information should be transcribed promptly after the end of the interview, and the duration of the interview was controlled to be between 30 ~ 50 min. The Colaizzi 7-step analysis method (27) was used to analyze the interview data, and categorical coding was performed to refine and summarize the interview results, and the study entries were increased to 35 entries.

### 2.4 Delphi expert correspondence

#### 2.4.1 Formulation of the questionnaire for expert correspondence

(1) Preface: including the purpose of the study, research methods, instructions for filling out the questionnaire, and the time and method of expert correspondence. (2) Expert opinion form, including the specific content of the indicators at all levels, the degree of importance, content validity judgment, and modification of the comment column. The importance of indicators at all levels adopts the Likert 5-level scoring method, from “unimportant” to “very important” to assign 1–5 points, and the content validity of indicators at all levels adopts the Likert 4-level scoring method, from “irrelevant” to “non-relevant,” and the content validity of indicators at all levels adopts Likert 4-level scoring method, from “non-relevant” to “non-relevant.” The content validity of indicators at all levels is assigned 1 ~ 4 points in order from “irrelevant” to “non-study related” by the Likert 4-level scoring method. (3) Experts’ questionnaire, including experts’ basic information, experts’ familiarity with the content of the survey, and the basis for judgment.

#### 2.4.2 Determining experts for correspondence

The purposive sampling method was used for expert correspondence. Expert inclusion criteria: (1) clinical nursing experts with undergraduate education or above, associate senior level or above, and 10 years or above of clinical nursing work in radiotherapy departments in tertiary-level A hospitals; (2) nursing management experts with undergraduate education or above, intermediate level or above, and 10 years or above of nursing management work in radiotherapy departments in tertiary-level A hospitals; (3) clinical medical experts with medical doctorate, and 10 years or above of nursing management work in tertiary-level A hospitals; and (4) clinical medical experts with medical doctorate, and 10 years or above of clinical nursing work in radiotherapy departments in tertiary-level A hospitals. A hospital engaged in clinical medical work in the radiotherapy department for 10 years or more, with the title of deputy senior grade or above; (4) highly motivated and willing participants in this study.

#### 2.4.3 Implementation of expert consultation

Correspondence questionnaires were distributed and collected by mail, and 2 rounds of expert consultation were implemented from January–February 2024 onwards. The entries were added or merged, deleted or modified, etc., based on the opinions of the expert correspondence consultation, and finally, 31 entries were formed (entry deletion criteria: entries with a mean score of importance <4, a coefficient of variation >0.25, or a full score rate <50%).

### 2.5 Pre-survey

Using the convenience sampling method, a pre-survey was proposed to be conducted from February 2024 to March 2024 for those who had been treated with nasopharyngeal carcinoma radiotherapy in the radiotherapy department of the First Affiliated Hospital of Guangxi Medical University. (1) Inclusion criteria: ① Pathohistologically and histologically confirmed diagnosis of nasopharyngeal squamous cell carcinoma; ② Age ≥ 18 years old; ③ All were in the first course of treatment; ④ All were treated with radiotherapy; ⑤ Carpenter’s score ≥ 90; ⑥ No major mental illnesses or disorders of consciousness in the past or at present; ⑦ Voluntarily participated in the study of the subject and signed written informed consent. (2) Exclusion criteria: ① Combined with other malignant tumors; ② Receiving anti-tumor related treatment before enrollment; ③ Having serious hearing impairment or communication disorders; ④ Combined with serious heart, lung and brain diseases. (3) Withdrawal criteria: ① Those who withdrew in the middle of the survey or whose condition changed; ② Those who filled out the questionnaire incompletely, with wrong or missing items.

The sample size was calculated according to the dimension with the highest number of entries in the pre-survey, i.e., 3 ~ 5 times the number of entries in that dimension (28). There were 10 entries in the preliminary scale of nutritional behavioral practice literacy, and 20% of invalid scales were considered, so the sample size was at least 36 cases.

### 2.6 Formal investigation

From April 2024 to December 2024, 245 patients with nasopharyngeal carcinoma radiotherapy who were initially treated in the radiotherapy department of the First Affiliated Hospital of Guangxi Medical University were collected as the study subjects. The inclusion and exclusion criteria of the study subjects were shown in the pre-survey. The sample size required for exploratory factor analysis or validation factor analysis was at least 200 cases (29), and at least 240 patients were needed to consider the 20% loss rate. To enhance the readability of the questionnaire statements and the reliability of the results, 10 of the entries were designed as reverse entries, adjusted for scoring, with higher scores indicating higher nutritional literacy in patients undergoing simultaneous radiotherapy for nasopharyngeal carcinoma.

### 2.7 Data collection method

The purpose and significance of the study were introduced to the patients by the researcher herself, and the paper Nutritional Literacy Assessment Scale for Nasopharyngeal Carcinoma Radiotherapy Patients was distributed after obtaining the consent of the patients and their families and signing the informed consent form. Patients completed the questionnaire independently, and if there were any questions, the entries were explained in a uniform language and patients were instructed to answer. For patients who had difficulty understanding or could not fill in the questionnaire by themselves, the researcher read out the content of the entries to them one by one without any suggestive language and recorded the patients’ choices. All the questionnaires were collected in time and the content was verified, and if there were any missing items, they were promptly supplemented and completed.

### 2.8 Statistical methods

Two members of the group double-checked the data entered, and the data were analyzed using SPSS 23.0 and AMOS 24.0 software. Quantitative data that conformed to normal distribution were expressed as mean ± standard deviation, and qualitative data were expressed as frequency and percentage (%). Item analysis was performed by the critical ratio method and correlation coefficient method, the validity test was performed by content validity and structural validity analysis, and the reliability test was performed by consistency reliability, folding reliability, and retest reliability analysis. *p* < 0.05 was taken as statistically significant.

#### 2.8.1 Item analysis method

(1) Critical ratio method: The first 27% and the last 27% of the patients’ questionnaire scores were taken as the high and low groupings, respectively, and the two independent samples t-test was used to delete the entries with critical ratio (CR) < 3.000 or *p* > 0.05 Correlation coefficient method: The correlation between the scores of each entry of the questionnaire and the total scores of the questionnaire was evaluated by Pearson correlation analysis, and correlation coefficients <0.4 or *p* > 0.05 were deleted for The entries with correlation coefficients <0.4 or *p* > 0.05 were deleted.

#### 2.8.2 Reliability and validity tests

##### 2.8.2.1 Validity test

(1) Content validity: According to the results of the expert correspondence, the item level content validity index (I-CVl) and the average questionnaire level content validity index (S-CVI) are calculated, and 1-CVI > 0.780, S-CVI > 0.8 indicate that the questionnaire has good content validity. -CVI > 0.8 indicates that the questionnaire has good content validity.
(2) Structural validity: When exploratory factor analysis was conducted, the KMO value and Bartlett’s spherical test were used to determine whether it was suitable for exploratory factor analysis. Principal component analysis and variance-maximizing orthogonal rotation were used to extract the common factors with eigenvalues >1. Factor loadings >0.4 and cumulative variance contribution >50% for each entry indicated good structural validity of the questionnaire, and AMOS 24.0 software was used to verify the goodness of fit of the dummy model for validation factor analysis (30). Validation factor analysis χ^2^/df < 3, root mean square error of approximation (RMSEA) < 0.08, goodness-of-fit index (GFI), comparative fit index (comparative fit index, CFI), incremental fit index (IFI), and normed Fit Index (NFI) > 0.80 indicate that the stability of the model is acceptable.

##### 2.8.2.2 Confidence test

(1) Internal consistency reliability: Cronbach’s alpha coefficient was used to assess the internal consistency of the questionnaire and the dimensions, and the overall Cronbach’s alpha coefficient of the questionnaire >0.8 indicated good internal consistency reliability;
(2) Folded-in-half reliability: the odd-even grouping method was used to divide all the items of the scale into two halves by the ordinal number, and Spearman-Brown was used to conduct the folded-in-half reliability analysis;
(3) Retest reliability: facilitate the selection of 245 cases in the formal survey after 2 weeks to issue the questionnaire again, using Pearson correlation analysis to calculate the correlation coefficient of the data of the two surveys, the correlation coefficient > 0.7 indicates that the questionnaire stability is good.

## 3 Results

### 3.1 Expert consultation outcomes

A two-round Delphi consultation was conducted with 15 experts from tertiary hospitals across four provinces (Guangxi: 8; Sichuan: 2; Guangdong: 3; Henan: 2). The expert panel comprised practitioners aged 45–55 years (49.33 ± 2.97) with 12–20 years (15.93 ± 2.43) of clinical experience, including 7 bachelor’s, 6 master’s, and 2 doctoral degree holders (3 intermediate, 8 associate senior, and 4 senior professionals).

Both consultation rounds achieved 100% response rates. The authority coefficients were 0.765 (Round 1) and 0.916 (Round 2), indicating high expert credibility. Kendall’s W concordance coefficients were 0.533 (*p* < 0.05) and 0.636 (*p* < 0.05) for respective rounds, with inter-round variation coefficients decreasing from 0.096–0.266 to 0–0.173.

Through iterative revisions:

(1) 6 items were eliminated (variation coefficient >0.25)
(2) 4 novel items were added:
  - NKCL-2: Knowledge of nutritional support pathways (diet→ONS → EN → PN) and transition criteria;
  - NKCL-3: Understanding routine nutritional screening (NRS-2002/PG-SGA) Importance;
  - NBPL-2: Competence in standardized dietary monitoring;
  - SAML-5: Application of oral lubricants to alleviate xerostomia;
(3) 2 items were merged:
  - AABSL-1: Integration of nutritional knowledge into sustainable dietary behaviors;
  - AABSL-7: Rejection of “starvation therapy” misconceptions;
(4) 3 items underwent terminological refinement

The final scale comprised 31 items across four dimensions:

- Nutritional Knowledge Cognition Literacy (7 items);
- Nutritional Behavioral Practice Literacy (8 items);
- Symptom Adaptive Management Literacy (7 items);
- Attitude and Belief Support Literacy (9 items).

### 3.2 Psychometric validation

#### 3.2.1 Content validity

The instrument demonstrated excellent content validity with:

- Item-level CVI (I-CVI) range: 0.80–1.00
- Scale-level CVI (S-CVI): 0.91

#### 3.2.2 Construct validity

Exploratory Factor Analysis (EFA):

- KMO measure: 0.872
- Bartlett’s test: χ^2^ = 3286.45, *p* < 0.001
- Four factors explained the 62.3% cumulative variance
- All factor loadings >0.45

Confirmatory Factor Analysis (CFA):

- χ^2^/df = 2.13
- RMSEA = 0.068
- GFI = 0.89, CFI = 0.92, IFI = 0.91, NFI = 0.88

#### 3.2.3 Reliability

- Overall Cronbach’s *α* = 0.93 (Subscales: 0.81–0.89)
- Split-half reliability: 0.85 (Spearman-Brown)
- Test–retest reliability (*n* = 245): ICC = 0.88

### 3.3 Survey implementation

- Pilot testing (*n* = 55): 100% valid response rate; 5 items linguistically optimized
- Formal survey (*n* = 250): 98% valid response rate (245/250), mean completion time = 20 ± 3.2 min

This rigorous validation process confirms the scale’s robustness for assessing nutritional literacy in nasopharyngeal carcinoma patients undergoing concurrent chemoradiotherapy.

#### 3.3.1 General information on patients with synchronized radiotherapy for nasopharyngeal cancer

A total of 245 patients with nasopharyngeal carcinoma undergoing concurrent radiotherapy and chemotherapy participated in this study, aged between 20 and 63 years old, with 157 male patients (64.1%) being the main group. Other general information can be found in Table 1.

**Table 1:** General information on patients with synchronized radiotherapy for nasopharyngeal cancer.

Projects	Classification	Number of people	Proportion (%)
Gender	Male	157	64.1
	Female	88	35.9
Age (years)	20 ~ 63	42.56 ± 12.98	
Marital Status	Married or divorced	176	71.8
	Unmarried	69	28.2
Career	Farmers	96	39.2
	Workers	80	32.7
	Staff	47	19.2
	Other	22	9.0
Education level	Primary School	59	24.1
	Secondary Schools	136	55.5
	College and above	50	20.4
Household income per capita (yuan)	<3,000	47	19.2
	3,000–6,000	133	54.3
	>6,000	65	26.5
Medical payment method	Resident Health Insurance	143	58.4
	Employee health insurance	102	41.6
Clinical Staging	≤ II	81	33.1
	III	164	66.9
BMI(kg/m^2^)	<18.5	143	58.1
	18.5–23.9	92	37.4
	24–27.9	10	4.1

#### 3.3.2 Validity assessment

##### 3.3.2.1 Content validity

The content validity indices were calculated using expert evaluation. The item-level content validity index (I-CVI) ranged from 0.872 to 1.000, and the scale-level content validity index (S-CVI) reached 0.932. These values exceeded the recommended threshold of 0.80 for I-CVI and 0.90 for S-CVI, confirming adequate content validity of the questionnaire.

##### 3.3.2.2 Construct validity

###### 3.3.2.2.1 Exploratory factor analysis (EFA)

The EFA revealed appropriate factorability of the data as evidenced by a Kaiser-Meyer-Olkin (KMO) measure of 0.898 and a statistically significant Bartlett’s test of sphericity (χ^2^ = 4194.673, *p* < 0.001). Principal component analysis with varimax rotation extracted four factors with eigenvalues >1, accounting for 62.3% of the total variance. The factor structure comprised:

(1) Nutritional Knowledge and Cognitive Literacy (NKCL)
(2) Nutritional Behavior and Practical Literacy (NBPL)
(3) Symptom Adaptation and Management Literacy (SAML)
(4) Attitude and Belief Support Literacy (AABSL)

**Figure 2:**
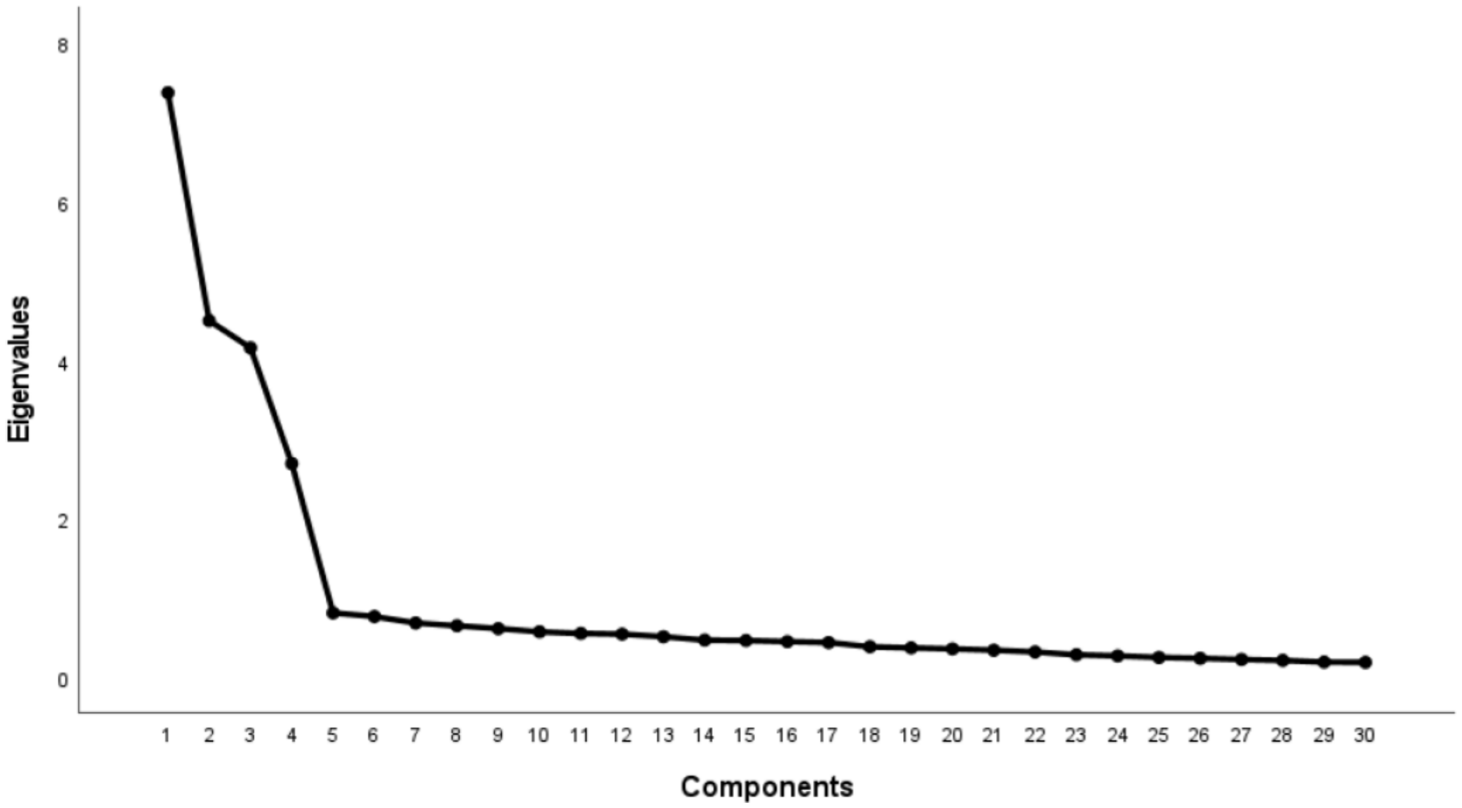
Scree plot.

The final 30-item scale included eight reverse-scored items: NKCL-2, NKCL-4, NBPL-2, NBPL-6, SAML-2, SAML-4, AABSL-2, and AABSL-6. All factor loadings exceeded 0.40, demonstrating satisfactory construct validity (Table 2). The scree plot results (see Figure 2).

**Table 2:** Factor loadings of the nutrition literacy assessment scale for nasopharyngeal carcinoma patients receiving concurrent chemoradiotherapy (NLA-CCRT).

Item Code	English version of scale items	NKCL	NBPL	SAML	AABSL
NKCL-1	I understand the daily requirements of macronutrients during CCRT (Protein: 1.2–1.5 g/kg/d; Calories: 30-35 kcal/kg/d)	0.252	0.398	0.233	0.841
NKCL-2	I recognize the stepped nutrition support pathway (Diet → ONS → EN → PN) and their transition criteria	0.210	0.39	0.223	0.824
NKCL-3	I acknowledge the importance of regular nutritional screening (NRS-2002, PG-SGA) for treatment planning	0.240	0.331	0.199	0.802
NKCL-4	I understand the rationale and precautions for frequent small meals during CCRT	0.369	0.33	0.209	0.752
NKCL-5	I know that appropriate regular exercise improves nutritional status	0.229	0.300	0.166	0.746
NKCL-6	I can identify ≥3 treatment-related nutritional risks (e.g., dysphagia from mucositis, anorexia from taste alterations)	0.201	0.363	0.222	0.687
NKCL-7	I know how to recognize malnutrition warning signs (e.g., 1-week weight loss >2% or albumin <35 g/L)	0.191	0.364	0.249	0.773
NBPL-1	I proactively acquire personalized nutritional information from healthcare providers/peers/authoritative sources	0.797	0.265	0.246	0.215
NBPL-2	I systematically document weight changes and dietary intake using standardized tools	0.785	0.304	0.316	0.387
NBPL-3	I promptly report acute treatment toxicities (nausea/vomiting/anorexia) affecting oral intake	0.755	0.214	0.246	0.258
NBPL-4	I adjust caloric intake based on weight trends (e.g., +300-500 kcal/d when weight loss occurs)	0.760	0.308	0.236	0.355
NBPL-5	I maintain adequate oral intake despite taste/olfactory alterations during CCRT	0.808	0.278	0.396	0.286
NBPL-6	I collaborate with healthcare providers for regular nutritional monitoring	0.821	0.233	0.292	0.201
NBPL-7	I implement symptom-specific nutritional strategies (e.g., liquid diet for VAS ≥ 4, tube feeding if swallowing efficiency<50%)	0.844	0.152	0.308	0.348
NBPL-8	I perform oral hygiene maintenance (brushing/rinsing) post-meal and pre-sleep	0.795	0.161	0.269	0.374
SAML-1	I routinely monitor and interpret key biochemical markers (Hemoglobin, prealbumin, transferrin, lymphocyte count)	0.302	0.802	0.227	0.205
SAML-2	I maintain body weight within normal range (BMI:18.5–23.9 kg/m^2^) through structured monitoring	0.303	0.774	0.284	0.314
SAML-3	I implement environmental optimization strategies (e.g., vomitus exposure prevention, HEPA filter use, cool-toned tableware to reduce nausea triggers)	0.298	0.804	0.326	0.241
SAML-4	I employ CCRT-specific dietary techniques (cool-temperature/soft-textured foods, small-bite pacing, avoiding acidic/irritating foods) to minimize mucosal irritation	0.275	0.782	0.24	0.346
SAML-5	I can apply oral moisturizers or artificial saliva to alleviate xerostomia for improved food intake	0.282	0.818	0.311	0.382
SAML-6	I adapt food flavors using lemon juice/spices to enhance appetite when experiencing taste alterations	0.307	0.795	0.309	0.396
SAML-7	I translate nutritional knowledge into sustainable dietary behavior modifications	0.317	0.833	0.252	0.406
AABSL-1	I perceive the necessity of regular professional nutrition counseling for NPC patients	0.323	0.207	0.721	0.295
AABSL-2	I believe nutritional status significantly impacts therapeutic outcomes and rehabilitation	0.318	0.257	0.735	0.224
AABSL-3	I consider regular nutritional monitoring crucial during CCRT	0.438	0.106	0.664	0.254
AABSL-4	I maintain that positive psychological status enhances nutritional metabolism during CCRT	0.388	0.134	0.693	0.217
AABSL-5	I value interdisciplinary nutrition communication with healthcare providers and peers	0.272	0.166	0.755	0.461
AABSL-6	I recognize the importance of family/community/peer-based nutritional support systems	0.386	0.219	0.715	0.215
AABSL-7	I affirm that evidence-based nutrition support does not promote tumor progression (dispelling the “starve the tumor” misconception)	0.260	0.229	0.781	0.187
AABSL-8	I acknowledge standardized nutrition support reduces treatment discontinuation rates	0.193	0.188	0.717	0.258
Eigenvalue		5.349	4.574	4.492	4.286
Variance contribution rate (%)		17.828	15.246	14.975	14.288
Cumulative variance contribution (%)		17.828	33.075	48.049	62.337

Bold values indicate primary factor loadings (>0.40). †Reverse-scored items. NPC, nasopharyngeal carcinoma; CCRT, concurrent chemoradiotherapy; ONS, oral nutritional supplements. Peers refer to fellow patients undergoing similar treatment regimens. NKCL, nutrition knowledge and cognitive literacy; NBPL, nutrition behavior and practical literacy; SAML, symptom adaptation and management literacy; AABSL, attitude and belief support literacy. Total variance explained: 62.337%.

###### 3.3.2.2.2 Confirmatory factor analysis (CFA)

**Figure 3:**
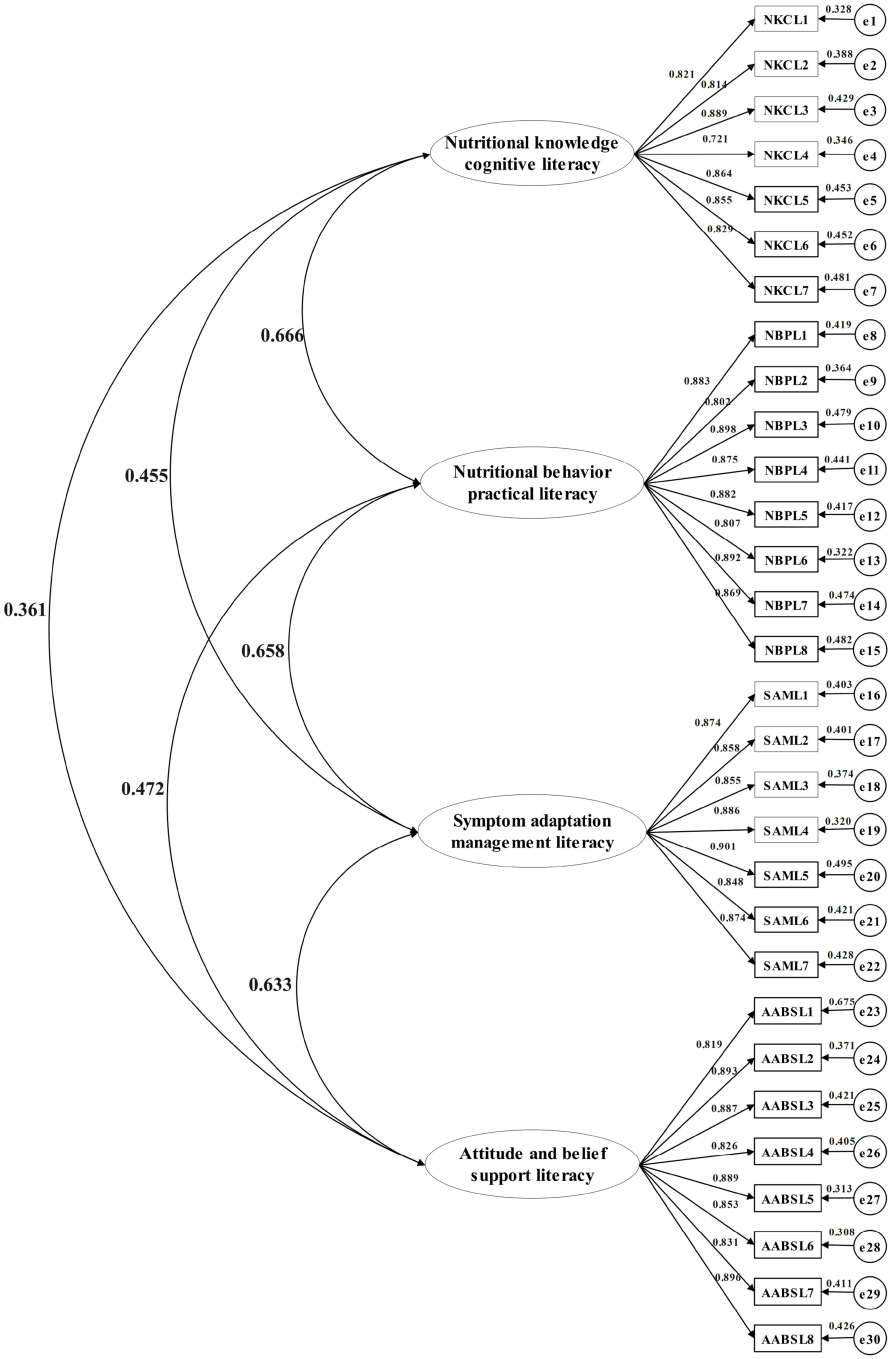
Confirmatory factor analysis chart.

A subsample of 245 nasopharyngeal carcinoma patients undergoing chemoradiotherapy was retested at 2-week intervals for CFA validation. The four-factor structure derived from exploratory analysis was examined using IBM SPSS AMOS 24.0. Model fit indices demonstrated excellent alignment with recommended thresholds: χ^2^/df = 1.155 (*p* = 0.085), RMSEA = 0.025 (90% CI: 0.000–0.043), CFI = 0.994, NFI = 0.956, IFI = 0.994, and GFI = 0.928. These results satisfied established psychometric criteria for structural validity (RMSEA <0.08, CFI > 0.90, NFI > 0.90) (1), confirming the hypothesized factor structure (see Figure 3).

#### 3.3.3 Reliability analysis

The nutritional literacy scale demonstrated robust psychometric properties through comprehensive reliability assessments (see Table 3).

**Table 3:** Reliability of the nutritional literacy scale for nasopharyngeal carcinoma patients undergoing simultaneous radiotherapy and chemotherapy.

Dimensions	Entry	Cronbach’s *α* coefficient	Spearman-Brown coefficient	Retest reliability
NKCL	7	0.863	0.835	0.901
NBPL	8	0.837	0.934	0.966
SAML	7	0.844	0.885	0.983
AABSL	8	0.810	0.893	0.954
Summary table	30	0.849	0.869	0.960

NKCL, nutrition knowledge and cognitive literacy; NBPL, nutrition behavior and practical literacy; SAML, symptom adaptation and management literacy; AABSL, attitude and belief support literacy.

## 4 Discussion

### 4.1 Reliability and validity of the nutrition literacy scale for NPC patients undergoing CCRT

This study developed a nutrition literacy scale tailored for NPC patients receiving concurrent chemoradiotherapy (CCRT) through rigorous methodological steps, including literature review, theoretical framework analysis, semi-structured interviews, Delphi expert consultation, and pilot testing. The final scale comprises four domains: “nutritional knowledge cognition,” “nutritional behavior practice,” “symptom adaptation management,” and“attitude-belief support.” Its design integrates evidence-based guidelines (31, 32) and addresses CCRT-specific symptoms (e.g., gastrointestinal toxicity, radiation-induced mucositis, dysphagia, and taste alterations), ensuring clinical relevance. To mitigate response bias, reverse-scored items were incorporated, and iterative refinements of item phrasing were conducted during qualitative interviews and pilot testing. Psychometric evaluations demonstrated robust measurement properties: (1) Content validity**: Item-level content validity index (I-CVI) ranged from 0.872 to 1.000, with a scale-level CVI (S-CVI) of 0.932. (2) Construct validity: Confirmatory factor analysis confirmed satisfactory model fit (CFI = 0.941, TLI = 0.926, RMSEA = 0.048), supporting the hypothesized four-factor structure. (3) Reliability: The total scale exhibited excellent internal consistency (Cronbach’s *α* = 0.849), with subscale α coefficients ranging from 0.810 to 0.863. Split-half reliability (total scale: 0.869; subscales: 0.835–0.934) and test–retest reliability (0.901–0.983) further confirmed temporal stability. These results validate the scale as a reliable and valid tool for early identification of nutritional risks in NPC-CCRT patients, facilitating timely interventions to optimize nutritional status and clinical outcomes.

### 4.2 Clinical and research implications of the nutrition literacy scale

Malnutrition affects 30–80% of NPC patients during CCRT, significantly worsened by treatment-related toxicities like radiation mucositis and dysphagia (32). While conventional nutritional assessments (e.g., PG-SGA, NRS-2002) provide valuable anthropometric and biochemical data, they lack critical dimensions: specifically evaluating patients’ acquisition of nutritional knowledge, capacity for dietary self-management, and skills to adapt to disease-specific challenges. Our multidimensional scale (encompassing Nutrition Knowledge Cognitive Literacy, Nutrition Behavioral Practice Literacy, Symptom Adaptation Management Literacy, and Attitudinal Belief Support Literacy) directly addresses these gaps. Its practical utility in clinical settings includes:

(1) Enhanced Risk Stratification & Early Intervention: Identifying patients with specific knowledge deficits or behavioral barriers at the outset allows clinicians to prioritize high-risk individuals for timely, targeted nutrition education before severe malnutrition develops.
(2) Dynamic Monitoring for Personalized Care: Tracking changes in nutrition literacy domains throughout the CCRT course enables healthcare teams to adapt interventions in real-time based on evolving patient needs and literacy levels, moving beyond static assessments.
(3) Precision Nutrition Implementation: Pinpointing specific literacy gaps (e.g., inadequate knowledge vs. poor practical skills vs. low self-efficacy) facilitates truly tailored strategies. This means directing resources effectively—such as offering knowledge-focused counseling to some patients, while providing hands-on skill-building (e.g., modified food preparation, symptom management techniques) or motivational support to others—thereby replacing inefficient “one-size-fits-all” approaches with evidence-based, individualized care (33).

## 5 Conclusion

The developed 30-item nutrition literacy scale demonstrates robust psychometric properties across four domains, offering a novel tool to assess knowledge, behaviors, symptom adaptation, and belief systems in NPC-CCRT patients. Its applications extend to risk screening, personalized education, and intervention efficacy evaluation. While validated in a CCRT-specific cohort, future multicenter studies should explore its generalizability to other cancer populations. Further refinement could incorporate digital health technologies (e.g., mobile app integration) to enhance real-time monitoring and patient engagement.

## Data Availability

The raw data supporting the conclusions of this article will be made available by the authors, without undue reservation.
